# Improved circuitry and post-processing for interleaved fast-scan cyclic voltammetry and electrophysiology measurements

**DOI:** 10.3389/frsip.2023.1195800

**Published:** 2023-11-29

**Authors:** Ashwin K. Avula, Abhinav Goyal, Aaron E. Rusheen, Jason Yuen, Warren O. Dennis, Diane R. Eaker, Joshua B. Boesche, Charles D. Blaha, Kevin E. Bennet, Kendall H. Lee, Hojin Shin, Yoonbae Oh

**Affiliations:** 1 Division of Engineering, Mayo Clinic, Rochester, MN, United States,; 2 Department of Neurologic Surgery, Mayo Clinic, Rochester, MN, United States,; 3 Medical Scientist Training Program, Mayo Clinic, Rochester, MN, United States,; 4 Neural Engineering Laboratories, Mayo Clinic, Rochester, MN, United States,; 5 Department of Biomedical Engineering, Mayo Clinic, Rochester, MN, United States

**Keywords:** fast-scan cyclic voltammetry, electrophysiological recording, post signal processing, dopamine, electrochemistry

## Abstract

The combination of electrophysiology and electrochemistry acquisition methods using a single carbon fiber microelectrode (CFM) in the brain has enabled more extensive analysis of neurochemical release, neural activity, and animal behavior. Predominantly, analog CMOS (Complementary Metal Oxide Semiconductor) switches are used for these interleaved applications to alternate the CFM output between electrophysiology and electrochemistry acquisition circuitry. However, one underlying issue with analog CMOS switches is the introduction of transient voltage artifacts in recorded electrophysiology signals resulting from CMOS charge injection. These injected artifacts attenuate electrophysiology data and delay reliable signal observation after every switch actuation from electrochemistry acquisition. Previously published attempts at interleaved electrophysiology and electrochemistry were able to recover reliable electrophysiology data within approximately 10–50 ms after switch actuation by employing various high-pass filtering methods to mitigate the observed voltage artifacts. However, high-pass filtering of this nature also attenuates valuable portions of the local-field potential (LFP) frequency range, thus limiting the extent of network-level insights that can be derived from *in vivo* measurements. This paper proposes a solution to overcome the limitation of charge injection artifacts that affect electrophysiological data while preserving important lower-frequency LFP bands. A voltage follower operational amplifier was integrated before the CMOS switch to increase current flow to the switch and dissipate any injected charge. This hardware addition resulted in a 16.98% decrease in electrophysiology acquisition delay compared to circuitry without a voltage follower. Additionally, single-term exponential modeling was implemented in post-processing to characterize and subtract remaining transient voltage artifacts in recorded electrophysiology data. As a result, electrophysiology data was reliably recovered 3.26 ± 0.22 ms after the beginning of the acquisition period (a 60% decrease from previous studies), while also minimizing LFP attenuation. Through these advancements, coupled electrophysiology and electrochemistry measurements can be conducted at higher scan rates while retaining data integrity for a more comprehensive analysis of neural activity and neurochemical release.

## 1 Introduction

In the past decade, combined electrochemistry (Echem) and electrophysiology (Ephys) experiments have provided a more comprehensive understanding of presynaptic and postsynaptic neuronal activity in the brain (Cheer et al., 2005). Electrophysiological recording enables monitoring of neuronal activity by measuring voltage fluctuations due to ion passage through neurons at the extracellular level (Li and Jasper, 1953). Action potentials, which are high frequency electrical events produced by individual neurons, as well as local-field potentials (LFPs), which are lower frequency (<100 Hz) synaptic events produced by clusters of neurons, can be measured by electrophysiological recording in this extracellular region (Gold et al., 2006; Buzsáki et al., 2012). However, electrophysiological recording is limited in its ability to quantify neurochemical transactions due to the absence of necessary voltage fluctuations. Conversely, fast-scan cyclic voltammetry (FSCV) is a well-established electrochemical method that measures evoked neurochemical release into the extracellular region by quantifying nanocurrents formed from displaced electrons during oxidation and reduction reactions (Cabrera et al., 2020; Venton and Cao, 2020). Performing coupled electrophysiological recording and FSCV at a single electrode *in vivo* allows for more sophisticated analysis of behavior with minimal damage to brain tissue (Covey and Cheer, 2019; Owesson-White et al., 2009; Su et al., 1990).

When performing coupled electrophysiological recording and FSCV using a single electrode, a significant amount of signal noise is introduced into the data stream when switching between acquisition modes. To address this issue, analog complementary metal oxide semiconductor (CMOS) switches are commonly used due to their low cost, power consumption, and low signal distortion (Corrigan and Goggin, 2004). However, CMOS switches are not without limitations, and one major problem is the presence of signal artifacts resulting from charge injection. When the gate voltage is alternated in a CMOS switch, charge flow between the gate-source and gate-drain capacitance induces a charge to the conduction channel, which appears as a low-frequency (<50 Hz) transient voltage spike in the carried signal with magnitudes on the order of microvolts (Wegmann et al., 1987; Yu, 2010). In the case of FSCV, a holding potential with a magnitude on the order of volts is initially applied to preconcentrate neurochemicals on the electrode surface (Puthongkham and Venton, 2020). Therefore, when switching from electrophysiological recording to FSCV, any charge-injected transients are dominated by the FSCV holding potential. However, electrophysiological signals are recorded with magnitudes on the order of microvolts, making charge injection artifacts more apparent when switching from FSCV to electrophysiological recordings (Shieh et al., 1987). Thus, voltage transients can delay and attenuate the measurement of reliable electrophysiological data after switching between modes.

The charge injection artifacts not only affect the measurement of electrophysiological signals but also cause a delay in the acquisition of reliable data, which in turn requires a slower repetition time in FSCV to increase the acquisition period and collect meaningful electrophysiological data. FSCV is ideally run at a 400 V/s scan rate with a repetition rate of 10 Hz to achieve a better signal-to-noise ratio, enhance neurochemical adsorption, and improve electrode sensitivity (Keithley et al., 2011; D. H. Kim et al., 2018; Venton and Cao, 2020). However, previous attempts at interleaved electrophysiological recording and FSCV used a 5 Hz cyclic voltammetry frequency and reported that full electrophysiological data recovery was not achieved until 50 ms (25% of the Ephys acquisition period) after the beginning of the acquisition period (Parent et al., 2017). Therefore, it is crucial to minimize the delay to allow for combined electrophysiological recording and FSCV at their respective optimal settings while maintaining high signal acquisition quality.

Efforts to combine electrophysiological recording and FSCV in previous studies used newer CMOS switches with low leakage, low charge transfer, low input capacitance, and low resistance to minimize the effects of charge injection (Parent et al., 2017). Additionally, various high-pass filters were implemented in post-processing to reduce the low-frequency voltage spikes in the recorded electrophysiological data (Takmakov et al., 2011; Belle et al., 2013; Parent et al., 2017). However, this filtering technique also attenuates physiologically relevant LFP frequency bands, such as Delta, Theta, Alpha, Beta, Gamma, High-Frequency, and Ripple, depending on the filter characteristics (Nukitram et al., 2021). The previous studies have mainly focused on recovering higher-frequency LFP bands and action potentials, but to gain a more comprehensive understanding of neurological events, it is important to retain as many LFP frequency bands as possible (Belitski et al., 2010).

This study proposes a solution to overcome the limitation of charge injection artifacts that affect electrophysiological data while preserving important lower-frequency LFP bands. To achieve this, the present study implements a voltage follower in the data acquisition circuitry and post-processing the data with curve-fit subtraction. This approach reduces the amplitude of injected voltage transient and shortens the delay until reliable electrophysiological data can be measured, all without the need for high-pass filtering. The outcome is the ability to combine electrophysiological recording and FSCV measurements with higher FSCV scan rates, while still retaining physiologically relevant LFP bands.

## 2 Materials and methods

### 2.1 Electrode fabrication

Carbon fiber microelectrodes (CFMs) were chosen for this study because of their superior sensitivity for interleaved applications. Although previous electrophysiology-only applications have utilized alternative electrode materials such as diamond or gold, CFMs exhibit higher sensitivity for FSCV applications compared to metal or diamond electrodes. Therefore, CFMs were the preferred electrode in this investigation due to their superior echem sensitivity and satisfactory electrophysiology sensitivity.

CFMs were fabricated using a previously established method (Oh et al., 2015, 2016; Barath et al., 2020). Briefly, a single carbon fiber (AS4, diameter = 7 μm; Hexcel, Stamford, CT) was inserted into a silica tube (ID = 20 μm, OD = 90 μm, 10 μm coat with polyimide; Polymicro Technologies, Phoenix, AZ) and sealed using epoxy resin (Clark et al., 2010). A nitinol extension wire (Nitinol #1, an alloy of nickel and titanium; Fort Wayne Metals, IN) was connected to the silica tubing and insulated with polyimide tubing (ID = 0.0089″, OD = 0.0134, WT = 0.00225; Vention Medical, Salem, NH). The carbon fiber tip was then trimmed to an approximate length of 50–70 μm. In addition, Teflon-coated silver wire (A-M systems, Inc., Sequim, WA) was chlorinated in saline to create Ag/AgCl reference and data input electrodes.

### 2.2 Experimental apparatus

**FIGURE 1:**
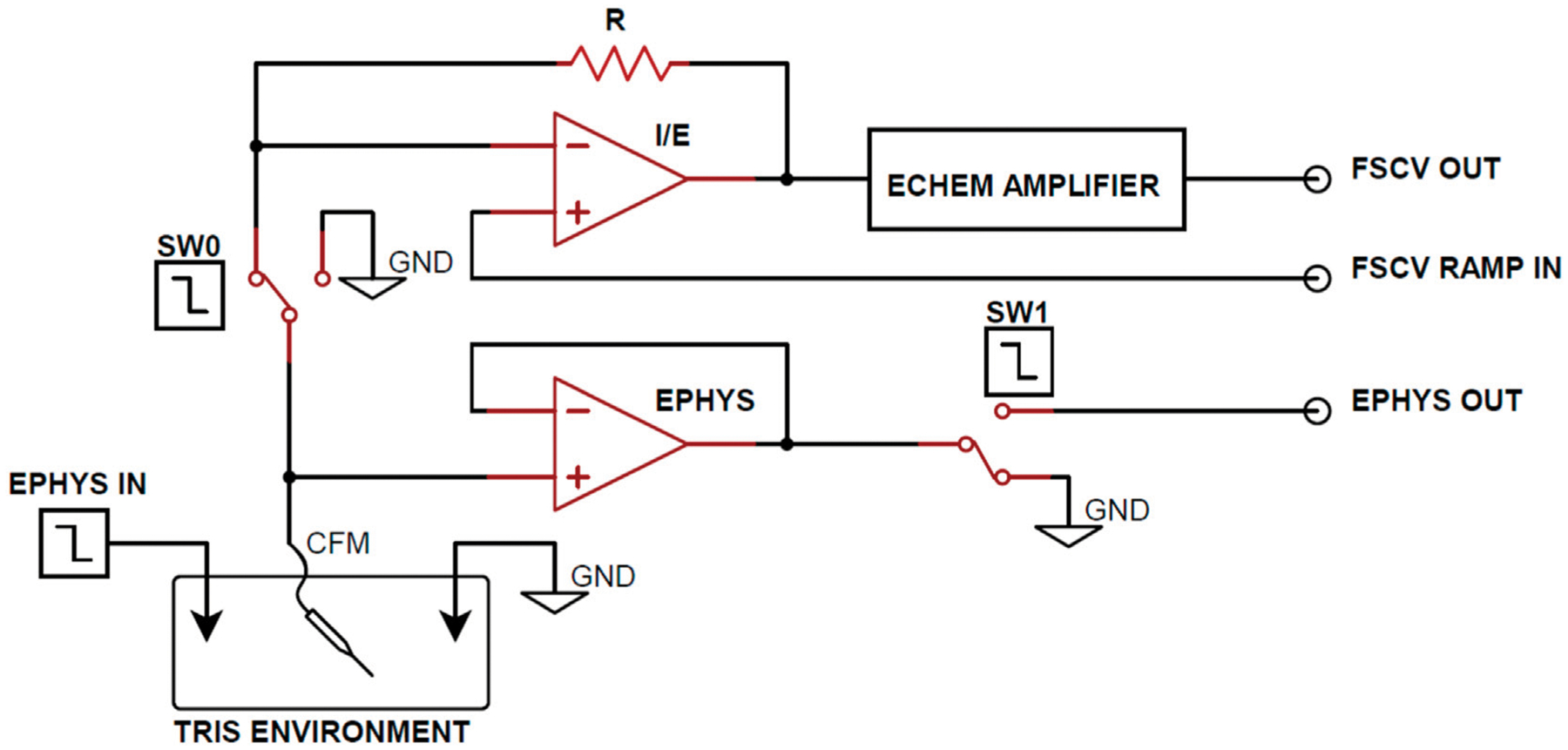
Functional circuit diagram for Ephys and FSCV acquisition. From the TRIS buffer *in vitro* environment, SW0 and SW1 alternate the working electrode output between the FSCV and electrophysiology acquisition circuitry. Arbitrary waveform generators are used to control the SW0 and SW1 enable and transmit electrophysiology data into the *in vitro* environment.

Figure 1 illustrates an *in vitro* experimental setup designed for coupled electrophysiological recording and FSCV. The apparatus consists of a CFM and a corresponding ground reference Ag/AgCl electrode submerged in a TRIS buffer and connected to both electrophysiological data and FSCV acquisition circuitry. Additionally, a data Ag/AgCl electrode is present in the buffer for the transmission of basal forebrain LFP recordings to simulate neuronal activity in the *in vitro* environment. Low charge injection and on-resistance MAX319 analog CMOS switches (Texas Instruments, Dallas, TX) were used to alternate the CFM output between electrophysiology and FSCV recording circuitry. Every 500 ms, the CFM output switches from electrophysiological recording to FSCV for 20 ms to perform only one acquisition mode at a time. The 480 ms delay between FSCV scans was used for testing purposes to increase the sampling window of electrophysiology recording for complete observation of the charge injection voltage artifact.

Operational amplifiers were employed in the FSCV circuitry to perform transimpedance and voltage offsetting and produce refined voltammograms at the FSCV Output. A voltage follower was implemented between CFM and the CMOS switch (SW1) in the electrophysiological recording circuitry to mitigate the effects of charge injection from the CMOS switch. Although the voltage follower maintains equal voltage on the input and output terminals, the high input resistance and low output resistance characteristics of the follower generates larger output current. This increased current flow dissipates injected charge from the CMOS device faster, resulting smaller voltage artifacts and decreased electrophysiology acquisition delay. Finally, electrophysiological signals were amplified to the millivolt scale at the Ephys output.

### 2.3 Input electrophysiology data

During the experimentation, open-source basal forebrain LFP recordings were continuously transmitted at the Ephys Data Input (Figure 1) to simulate *in vitro* neural activity, using a Keysight 33522B arbitrary waveform generator (Keysight, Santa Rosa, CA). To increase the signal-to-noise ratio, the LFP recordings were sent with a 1 mVpp amplitude. This approach was adopted to maintain consistency with *in vivo* neural signals and enhance the relevance of the *in vitro* experimentation (Nair et al., 2017a; 2017b).

### 2.4 Data acquisition

#### 2.4.1 Electrophysiology

The electrophysiological data was collected and amplified to the millivolt scale at the Ephys Ouput (Figure 1) at a sample rate of 12.5 kHz using an Intan RHD2000 USB interface board (Intan Technologies, Los Angeles, CA). The data was stored on a PC base-station for analysis. Finally, MATLAB (MathWorks Inc., Natick, MA) was used for the post-processing of recorded electrophysiology signals.

#### 2.4.2 Fast-scan cyclic voltammetry

To perform FSCV, a triangular waveform with a peak potential of 1.3 V and a holding potential of −0.4 V was applied at a repetition rate of 2 Hz with a scan rate of 400 V/s. FSCV voltammograms were recorded using WincsWare (Mayo Clinic, Rochester, MN) (Shin et al., 2019). This lower repetition rate was chosen for better analysis of the injected voltage artifacts, and the minimization of the artifacts will allow for the use of faster repetition rates when performing coupled FSCV and electrophysiology recording. It should be noted that while FSCV was used for the purpose of coupled FSCV and electrophysiological recording, charge injection post-processing was not necessary as previously discussed.

#### 2.4.3 Synchronizing acquisition circuitry

To guarantee accurate synchronization between FSCV and electrophysiological acquisition, a Keysight 33522B arbitrary waveform generator was utilized to enable the switches. This 2-channel device generated a 20 ms enable signal at 2 Hz to SW0 for FSCV acquisition and an inverted enable signal to SW1 for electrophysiological acquisition. This configuration ensured that only one acquisition circuitry line would be connected to the *in vitro* environment and CFM at a time.

### 2.5 Data analysis and post-processing

Figures and statistical analyses were generated using MATLAB and GraphPad Prism (GraphPad Software Inc., Boston, MA), respectively. One-way ANOVA and paired t-tests were used for statistical analysis. Mean ± standard error of the mean (SEM) values are presented for an n-number of electrodes.

#### 2.5.1 Voltage artifact characterization and removal

Previous studies for interleaved FSCV and electrophysiological recording have employed various filtering methods to reduce noise and attenuate transient voltage artifacts from recorded electrophysiological data (Takmakov et al., 2011). However, these methods have a drawback of attenuating parts of the LFP spectrum, causing a loss of physiologically relevant electrophysiological data. To address this, this study utilizes single-term exponential regression to characterize the observed resistive-capacitive (RC) transient voltage artifacts (Gómez-Aguilar et al., 2017). The modeled voltage artifact is then subtracted from the original electrophysiological output to recover the desired signal (Figure 1).

#### 2.5.2 Quantification of acquisition delay due to charge injection voltage artifacts

As explained earlier, charge injection creates a transient voltage spike that delays the recovery of reliable electrophysiological data when switching from FSCV acquisition. This delay is referred to as the “settling time” since the electrophysiological data is only reliable once the voltage artifact settles to the baseline voltage. To quantify the settling time, a thresholding function was used to determine when the average value of the voltage artifact had decayed to below one standard deviation of the input electrophysiology data after switch actuation. Thus, by comparing different post-processing methods by their settling times, the most optimal post-processing method for electrophysiology data recovery can be determined.

### 2.6 Validation of interleaved FSCV and electrophysiology

For *in vitro* validation, a dopamine flow injection system was utilized (D. H. Kim et al., 2014). A CFM was positioned at the center of an acrylic chamber that was connected to a flanged fluid line (BOLA, Germany) through a switching valve (Rheodyne MX series II, IDEX Health & Science, United States). A syringe pump (Harvard Apparatus, Holliston, MA) was employed to maintain a flow rate of 2 mL per minute. To assess the functionality of FSCV, 10-s boluses of 100 nM dopamine were intermittently injected into the CFM during the continuous injection of TRIS buffer.

## 3 Results

### 3.1 Effect of voltage follower in electrophysiology acquisition circuitry

**FIGURE 2:**
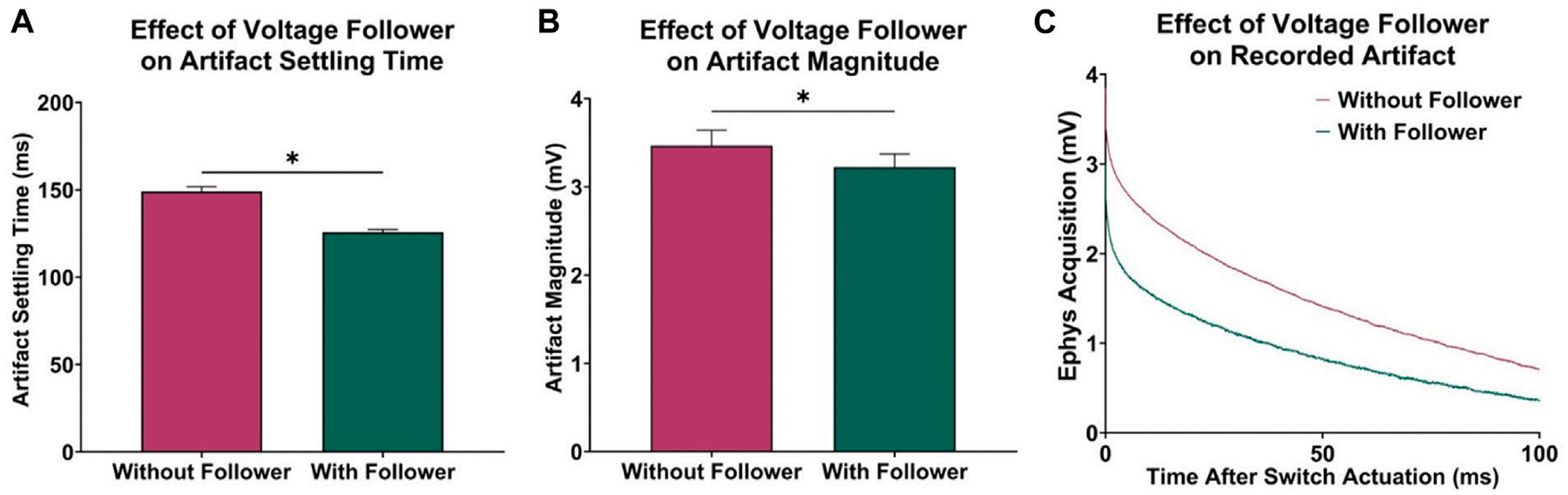
Effect of voltage follower in electrophysiology acquisition circuitry. **(A)** Observed decrease in acquisition delay with the addition of the voltage follower (*n* = 5 electrodes, paired t-test, *p* < 0.0001). **(B)** Observed decrease in initial magnitude of voltage artifact (*n* = 5 electrodes, paired t-test, *p* = 0.0003). **(C)** Sample recordings at the electrophysiology output, demonstrating the effect of the voltage follower on the transient voltage artifact.

In previous attempts to combine electrophysiological recording and FSCV, CMOS analog switches were used to minimize charge injection in the acquisition circuitry by optimizing for low leakage and charge transfer. However, to further improve this approach, a voltage follower was added to the electrophysiological circuitry between CFM and SW1 to minimize the effects of charge injection. The low output impedance characteristic of the voltage follower allowed for an increased current flow to the CMOS switch, resulting in a faster dissipation of any injected charge after switch actuation. To test this, data was recorded with and without the voltage follower, and the transient voltage artifact was observed by grounding the electrophysiological input waveform generator. Using paired t-tests, it was determined that the addition of the voltage follower decreased in recovery delay of 23.35 ± 0.54 ms (*n* = 5 electrodes, *p* < 0.0001, Figure 2A) and reduced the overall voltage artifact magnitude by 242.57 ± 24.27 μV (*n* = 5 electrodes, *p* = 0.0003, Figure 2B). Figure 2C shows the recorded charge injection voltage artifact (millivolt scale) during the first 100 ms of the electrophysiological data acquisition period after switch actuation, which dominates the signal (microvolt scale) until it settles back to the 0 V baseline, thus delaying the measurement of reliable electrophysiology data.

### 3.2 Effect of charge injection characterization and subtraction

In addition to artifact reduction from the implemented voltage follower in hardware, the curve characterization and subtraction method presented in this paper better minimized the voltage artifacts in recorded electrophysiological data. Before post-processing, electrophysiology data is recovered 125.80 ± 0.39 ms after switch actuation (Figure 2).

**FIGURE 3:**
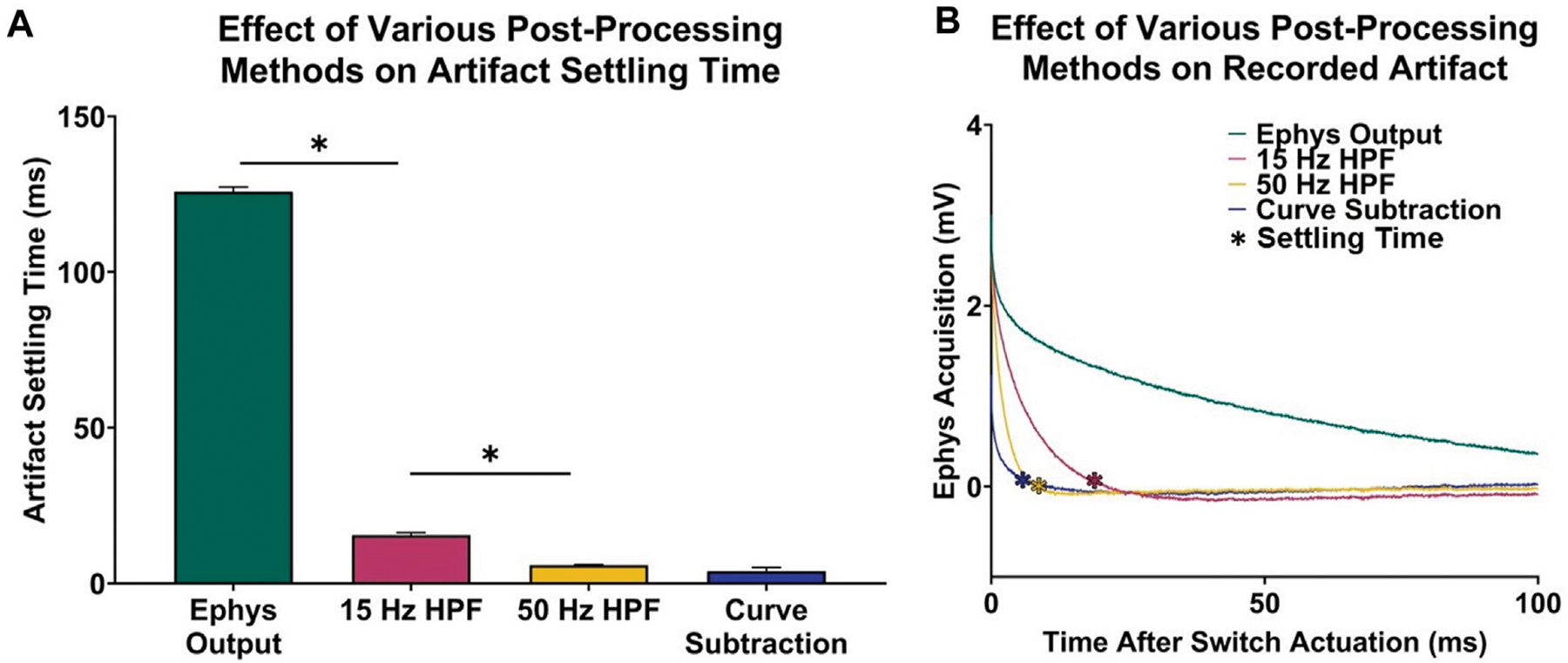
Voltage artifact settling time–electrophysiology acquisition delay–for various post-processing methods. **(A)** Settling times for selected signals. **(B)** Respective recordings portraying difference in transient voltage artifact for each signal type.

A one-way ANOVA demonstrated significant differences in artifact settling time among the different post-processing methods considered (*n* = 5 electrodes; *p* < 0.0001). Figures 3A, B demonstrates that a third-order high-pass filter with a cutoff frequency of 15 Hz resulted in a statistically significant 110.30 ± 0.43 ms decrease in acquisition delay (*n* = 5 electrodes, paired t-test, *p* < 0.0001), which is comparable to the signal recovery analysis performed by Parent et al. (2017) who were able to recover most of the electrophysiological data spectrum within 50 ms after switch actuation. Furthermore, increasing the filter cutoff frequency leads to faster artifact settling times, as a 50 Hz high-pass filter resulted in a 9.64 ± 0.21 ms decrease in delay when compared to a 15 Hz high-pass filter (*n* = 5 electrodes, paired t-test, *p* < 0.0001). To compare the difference in settling time between high-pass filtering and voltage artifact characterization and subtraction, the artifact was modeled by performing single-term exponential regression on each electrophysiology acquisition period. The exponentials were then subtracted from the original electrophysiology recording, resulting in the recovered electrophysiology data. Ultimately, this method results in a 11.57 ± 0.24 ms decrease in acquisition delay when compared to a 15 Hz high-pass filter (*n* = 5 electrodes, paired t-test, *p* < 0.0001).

**FIGURE 4:**
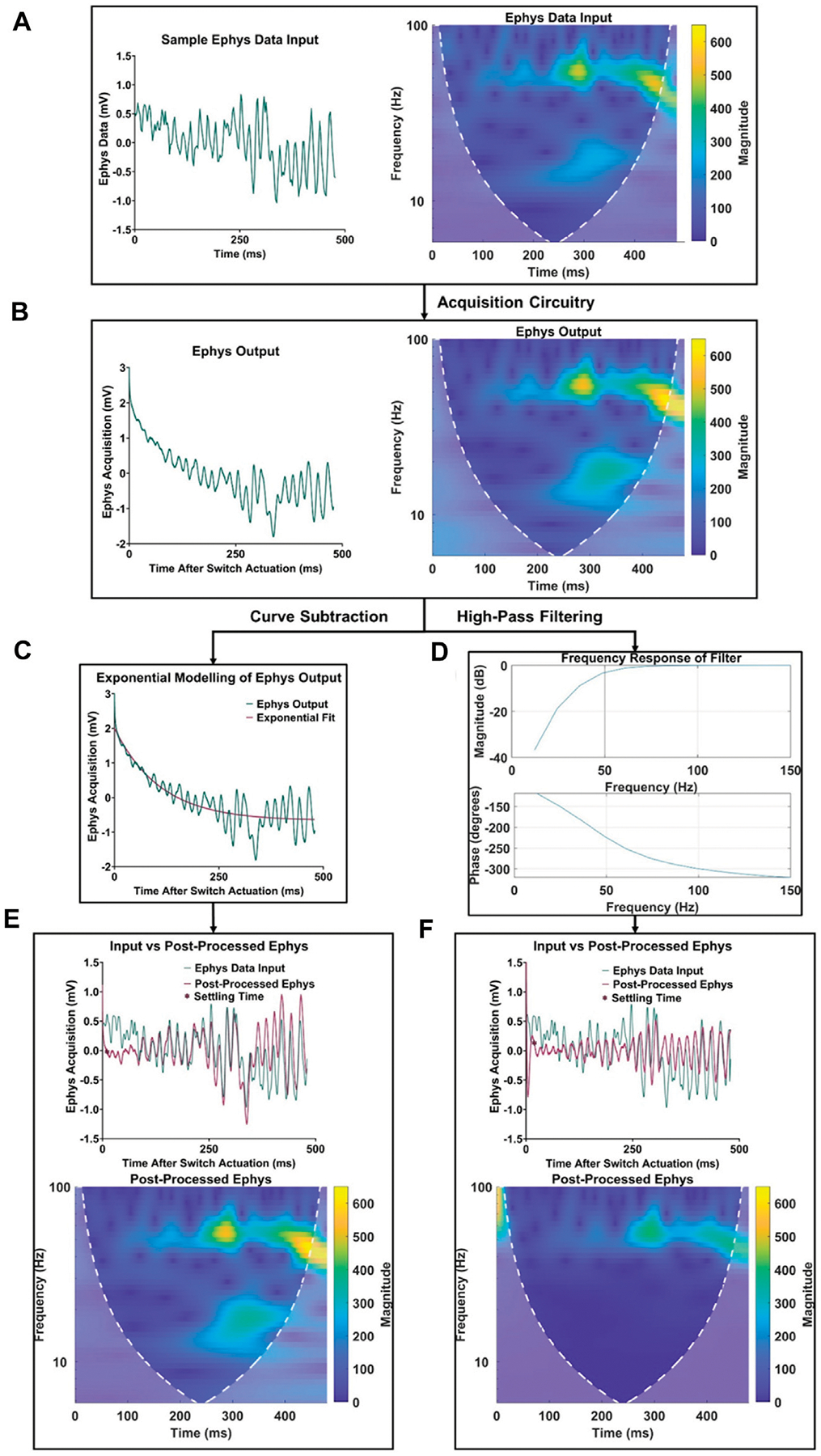
Comparison of electrophysiology data retention using high-pass filtering and the proposed curve characterization method. **(A)** Original sample signal input and its frequency spectrum. **(B)** Signal recording and frequency spectrum at the electrophysiology output. **(C)** Depiction of curve characterization and subtraction process. **(D)** Depiction of third order high-pass filter with a cutoff frequency of 50 Hz. **(E)** Signal comparison and frequency spectrum of post-processed electrophysiology data using the proposed curve characterization method. **(F)** Signal comparison and frequency spectrum of post processed electrophysiology data using a high-pass filtering method.

In addition to the improvements in acquisition delay, Figure 4 highlights the significant difference in signal quality between traditional high-pass filtering and the proposed characterization and subtraction method. The frequency spectrum between the electrophysiological data (Figure 4A) and output (Figure 4B) remained largely similar, except for the addition of low-frequency content within the first 100 ms in the electrophysiological recording output spectrogram due to the voltage artifact. After applying the respective post-processing methods (Figures 4C, D) to the electrophysiological output, it was observed in the time and frequency domain that the characterization and subtraction method better attenuates the voltage artifact while preserving more of the valuable lower-frequency electrophysiological content (Figures 4E, F).

### 3.3 Validation of interleaved FSCV and electrophysiology

**FIGURE 5:**
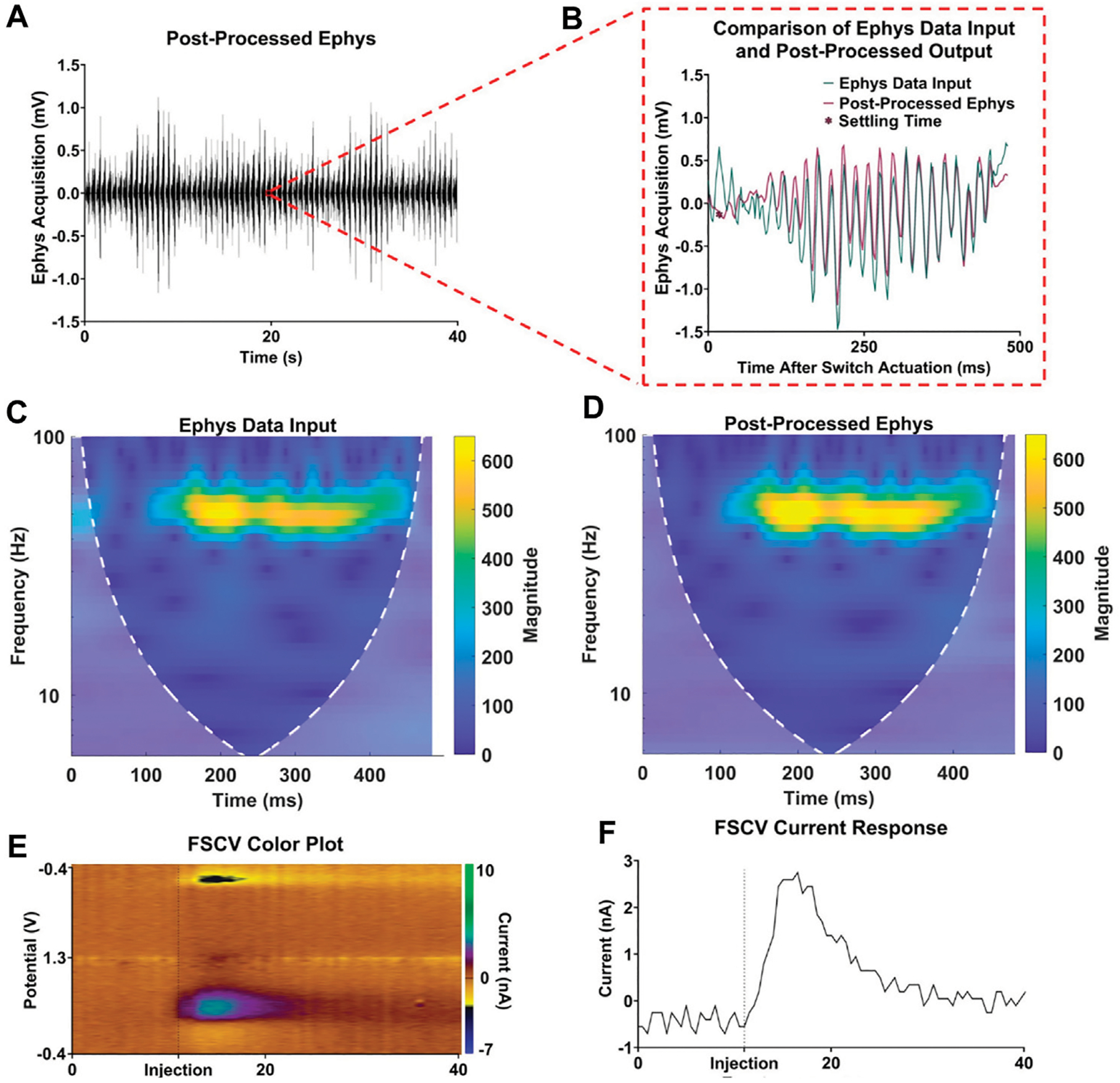
Validation of interleaved FSCV and electrophysiological recording. **(A)** Sample 40-s electrophysiology acquisition. **(B)** Single electrophysiology acquisition period comparison input and post-processed electrophysiology data. **(C,D)** Respective frequency spectra of input and post-processed electrophysiology data. **(E,F)** FSCV color plot and current response for a dopamine injection event at t = 11 s.

The *in vitro* apparatus was further validated by incorporating a flow cell system, which was used to periodically inject boluses of dopamine for FSCV measurement. This system enabled a more comprehensive assessment of interleaved electrophysiological recording and FSCV acquisition. As depicted in Figure 5, the techniques proposed here demonstrated minimal signal loss while retaining FSCV dopamine sensitivity. Figure 5A displays a 40-s post-processed electrophysiological recording with 80 electrophysiological acquisition periods. Within this 40-s recording, Figure 5B exhibits a single electrophysiological acquisition period with the corresponding data input and post-processed output, highlighting signal retention in the time domain. Additionally, Figures 5C, D show the preservation of frequency content in the sample data from Figure 5B. Finally, Figures 5E, F display the corresponding 40-s sample FSCV recording with a dopamine injection event at t = 11 s, revealing the reliable performance of the coupled FSCV and electrophysiological recording with minimal acquisition delay and signal attenuation.

## 4 Discussion

This paper details the improved circuitry and post-processing techniques employed to minimize the effects of CMOS charge injection during interleaved FSCV and electrophysiological recordings. These improvements include the addition of an operational amplifier voltage follower to the acquisition circuitry, as well as the characterization and subtraction of charge injection artifacts. These refinements result in electrophysiological recordings with minimal acquisition delay and minimal attenuation of lower frequency LFP bands, allowing for optimal scan rates during FSCV acquisition for interleaved applications, while preserving important low-frequency neural phenomena.

Traditionally, voltage followers, also known as unity gain buffer amplifiers, have been used to transfer voltage from high impedance circuits to lower impedance circuits. This is because voltage followers can provide significant current gain while maintaining voltage across a significant impedance reduction, making them ideal for measuring fast-changing signals from high impedance sources like electrode probes in bioelectronics (De Man et al., 1977). For this reason, voltage followers have been used in electrophysiological data acquisition circuitry to capture fast-changing biological potentials (Allen and Toyama, 1973; Mabilde, 1974). Previous attempts to incorporate voltage followers in interleaved electrophysiological recording and FSCV acquisition circuitry resulted in the introduction of charge injection artifacts into the recorded electrophysiological signals. This issue occurred due to the placement of the voltage follower after CMOS switches, which alternate the working electrode between FSCV and electrophysiological recording circuitry (Takmakov et al., 2011; Parent et al., 2017). While charge injection from CMOS switches is unavoidable in interleaved applications, placing the voltage follower before the CMOS device was shown to decrease the effect of injected charge on the recorded Ephys signal. Models of charge injection from CMOS devices have shown that the switch charge injection effect is functionally dependent on the driving force, thus the additional current gain from the voltage follower to the CMOS switch influences the presence of injected charge (Shieh et al., 1987). Further improvements to acquisition circuitry could include circuit techniques such as gain boosting or switch bootstrapping to overcome the undesired effects of charge injection and minimize its prominence in recorded Ephys signals (Razavi, 2015; Tiwari and Talwekar, 2020).

More recently, LFP analysis have been pursued to aid deep-brain stimulation (DBS) research and treatment of neurological disorders such as Parkinson’s disease (Lempka and McIntyre, 2013). Previous attempts at interleaved FSCV and electrophysiological data acquisition systems to aid DBS research experienced the presence of charge injection voltage artifacts in electrophysiological recordings, limiting the amount of electrophysiological analysis that could be performed and forcing the use of lower FSCV repetition rates to compensate for the settling time of the transient voltage artifact (Parent et al., 2017). High-pass filtering of about 10 Hz was used in these interleaved applications to attenuate the recorded artifacts and recover electrophysiological data earlier in the acquisition period. Additionally, high-pass filtering of this nature has been prominent in independent electrophysiological implementations that experienced transient artifacts resulting from CMOS devices (K. Kim et al., 2020). However, while filtering can minimize the effects of low-frequency charge injection artifacts, it also attenuates LFP delta, theta, alpha, and beta LFP bands (<30 Hz) that are imperative for DBS studies on motor symptoms and disorders such as Parkinson’s (Kajikawa and Schroeder, 2011; Lempka and McIntyre, 2013). To overcome this, exponential modeling was implemented to characterize the transient nature of the charge-injected voltage artifacts (Dei and Valle, 2006). Characterization and subtraction allowed for more accurate removal of the introduced transient artifact while preserving the valuable lower-frequency LFP bands. As a result, interleaved applications can more accurately record LFP data while maintaining a more optimal FSCV scan rate. These improvements to acquisition technology facilitate a more extensive analysis of presynaptic and postsynaptic neuronal activity for the next-generation of studies in drug addiction and mental disorders (Fitzgerald and Watson, 2019; Kolpakova et al., 2022).

One issue that may impact interleaved applications is the irregularity of charge injection and the inconsistency of recorded voltage artifacts. Charge injection is directly related to the on-resistance of CMOS devices. Therefore, varying impedances on the CFM input from different experimental environments can affect the transient nature of the charge injected artifact (Tiwari and Talwekar, 2020). For this reason, the decision was made to characterize and subtract artifacts individually after every switch actuation. While this method accurately removes artifacts, it requires more computationally intensive curve characterization and subtraction. This level of signal processing may not be feasible for certain real-time applications or mobile acquisition devices. For interleaved applications that require instant real-time display and analysis of interleaved data, curve characterization may be applied once initially and used as a general model to subtract from all artifacts recorded after switch actuation. This generalization of the charge injection characterization will significantly reduce computational intensity, offering real-time capabilities at the expense of electrophysiological signal quality due to artifact inconsistencies.

The advancements and methodologies introduced in this paper should be further developed and implemented in interleaved technologies, if feasible, to leverage their combined strengths and overcome individual limitations. For example, higher sampling rates in electrophysiology acquisition can enhance resilience against ambient noise during recordings, in addition to capturing high-speed action potentials (Jaw, 2001). This heightened resolution has the potential to minimize distortion in electrophysiology data during post-processing. Moreover, the incorporation of digital signal processing devices in the acquisition circuitry can facilitate real-time data processing and artifact removal, thus amplifying the impact of this solution in commercial applications. Finally, the use of carbon nanotube-based electrodes has proven to result in lower electrode impedance, increased neuronal adhesion, and a higher concentration of neurochemicals on the electrode surface due to their rough nanostructure (Mazzatenta et al., 2007). While these electrodes are more challenging to fabricate than the carbon fiber electrodes used in this study, their advantages could potentially lead to higher signal-to-noise ratios, faster scan rates, and increased biocompatibility for future interleaved applications (Asis et al., 2010).

Although no *in vivo* experiments were conducted in this study, the transmission of *in vivo* LFP data within the *in vitro* environment provided an adequate simulation of neural activity for testing the post-processing methods. Considering that the circuit adjustments were applied to existing *in vivo* acquisition circuitry and the post-processing techniques can be implemented on any interleaved electrophysiological data, it is expected that future *in vivo* applications will benefit from the findings presented in this paper. With the improvements introduced in this paper and the availability of next-generation tools, interleaved FSCV and electrophysiology applications will enable the recording of more precise and desirable data, leading to a deeper understanding of neuronal activity in the brain.

## 5 Conclusion

This paper proposes solutions to reduce the impact of CMOS charge injection on electrophysiological recordings during interleaved FSCV applications. Charge injection from data acquisition circuitry causes voltage artifacts that can affect the accuracy of recorded electrophysiological data. To minimize this effect, the paper suggests the use of an operational amplifier voltage follower in the acquisition circuitry, as well as the application of post-processing techniques, such as single-term exponential modeling, to characterize and subtract charge injection artifacts. These improvements enable the acquisition of both electrophysiological and FSCV data at optimal scan rates and minimal acquisition delay, making it possible to study presynaptic and post-synaptic neural activity more effectively. The proposed hardware and post-processing advancements aim to provide deeper insights into neural activity and brain function and the integration of on-board digital signal processing devices will enhance the suitability of acquisition technologies for neurological studies.

## Data Availability

The raw data supporting the conclusion of this article will be made available by the authors, without undue reservation.
